# Using Electronic Health Record Data to Determine the Safety of Aqueous Humor Liquid Biopsies for Molecular Analyses

**DOI:** 10.1016/j.xops.2024.100517

**Published:** 2024-03-19

**Authors:** Julian Wolf, Teja Chemudupati, Aarushi Kumar, Joel A. Franco, Artis A. Montague, Charles C. Lin, Wen-Shin Lee, A. Caroline Fisher, Jeffrey L. Goldberg, Prithvi Mruthyunjaya, Robert T. Chang, Vinit B. Mahajan

**Affiliations:** 1 Department of Ophthalmology, Spencer Center for Vision Research, Byers Eye Institute, Stanford University, Palo Alto, California; 2 Molecular Surgery Laboratory, Stanford University, Palo Alto, California; 3 Faculty of Medicine, Eye Center, Medical Center, University of Freiburg, Freiburg, Germany; 4 Department of Radiation Oncology, Stanford University, Palo Alto, California; 5 Veterans Affairs Palo Alto Health Care System, Palo Alto, California

**Keywords:** Aqueous humor liquid biopsy, Electronic health records, Free-text search, Intraoperative complications, Precision medicine

## Abstract

**Purpose:**

Knowing the surgical safety of anterior chamber liquid biopsies will support the increased use of proteomics and other molecular analyses to better understand disease mechanisms and therapeutic responses in patients and clinical trials. Manual review of operative notes from different surgeons and procedures in electronic health records (EHRs) is cumbersome, but free-text software tools could facilitate efficient searches.

**Design:**

Retrospective case series.

**Participants:**

A total of 1418 aqueous humor liquid biopsies from patients undergoing intraocular surgery.

**Methods:**

Free-text EHR searches were performed using the Stanford Research Repository cohort discovery tool to identify complications associated with anterior chamber paracentesis and subsequent endophthalmitis. Complications of the surgery unrelated to the biopsy were not reviewed.

**Main Outcome Measures:**

Biopsy-associated intraoperative complications and endophthalmitis.

**Results:**

A total of 1418 aqueous humor liquid biopsies were performed by 17 experienced surgeons. EHR free-text searches were 100% error-free for surgical complications, >99% for endophthalmitis (<1% false positive), and >93.6% for anesthesia type, requiring manual review for only a limited number of cases. More than 85% of cases were performed under local anesthesia without ocular muscle akinesia. Although the most common indication was cataract (50.1%), other diagnoses included glaucoma, diabetic retinopathy, uveitis, age-related macular degeneration, endophthalmitis, retinitis pigmentosa, and uveal melanoma. A 50- to 100-μL sample was collected in all cases using either a 30-gauge needle or a blunt cannula via a paracentesis. The median follow-up was >7 months. There was only one minor complication (0.07%) identified: a case of a small tear in Descemet membrane without long-term sequelae. No other complications occurred, including other corneal injuries, lens or iris trauma, hyphema, or suprachoroidal hemorrhage. There was no case of postoperative endophthalmitis.

**Conclusions:**

Anterior chamber liquid biopsy during intraocular surgery is a safe procedure and may be considered for large-scale collection of aqueous humor samples for molecular analyses. Free-text EHR searches are an efficient approach to reviewing intraoperative procedures.

**Financial Disclosure(s):**

Proprietary or commercial disclosure may be found in the Footnotes and Disclosures at the end of this article.

Paracentesis of the anterior chamber (AC) has been used for decades to therapeutically lower intraocular pressure and remove pathologic tissues such as blood or inflammatory cells. AC biopsies are an important diagnostic tool for patients with uveitis,^1^ and, more recently, aqueous humor (AH) liquid biopsies are used for molecular testing in research. They allow the capture of locally enriched fluids containing thousands of molecules from healthy and diseased ocular tissues, which can be detected using a variety of molecular assays, including proteomics, metabolomics, and DNA sequencing. These tests offer the potential to understand disease mechanisms in living humans and to identify novel diagnostic and therapeutic strategies. Specifically, AH proteomics has been shown to predict the treatment response of patients with neovascular age-related macular degeneration and diabetic macular edema, 2 of the most frequent blinding diseases.^2^^,^^3^ Analyzing cell-free DNA in AH has demonstrated potential to identify somatic genomic alterations in retinoblastoma, the most common eye cancer in childhood^4^^,^^5^ and can help to specify the underlying pathogen in patients with intraocular infections.^1^^,^^6^ In addition, AH metabolomics studies have identified new potential biomarkers and therapeutic targets for diabetic retinopathy and glaucoma.^7^^,^^8^ We recently found that state-of-the art techniques for increased AH proteomic throughput combined with single-cell RNA sequencing of ocular tissues, allow cell level analyses in living patients, even in tissues such as the retina that are not amenable for direct tissue biopsies.^9^

Aqueous humor samples are routinely collected at the slit lamp in clinic mainly for diagnostic purposes and previous comparatively small studies have shown this to be a safe procedure in this setting.^10^, ^11^, ^12^, ^13^ Anterior chamber biopsies are also increasingly collected during human clinical trials to investigate target engagement and biomarkers associated with clinical outcomes.^14^ Large-scale collection of AH specimens can be performed at the beginning of intraocular procedures, including cataract, glaucoma, corneal, or vitreoretinal surgeries, which are among the most frequently performed surgeries worldwide. However, although part of a surgery, the AH biopsy may theoretically cause specific additional complications, such as corneal trauma, iris trauma, lens touch, hyphema, or suprachoroidal hemorrhage from hypotony.

Studies reviewing surgical procedures and their complications are challenging at large scale because coding in surgical reports frequently reflects only the general surgical procedures and not the complications. The use of International Classification of Diseases codes may be problematic to accurately assess diagnoses or surgical outcomes, since not all ocular conditions or surgical complications have a specific code, and clinicians may upcode or downcode certain conditions or may not take the time to code all conditions. A recent study found that only about 50% of cases with lens pathology could be detected by analyzing International Classification of Diseases billing codes, whereas a free-text search-based algorithm identified >95% of the cases.^15^ Analyzing surgical complications at large scale requires tedious manual review of operative and postoperative notes, especially since different surgeons may use different language to describe intraoperative events. The requirement to manually read surgical reports can significantly limit the number of cases that can be reviewed. It has previously been shown that electronic health record (EHR) models that combine structured data with free-text searches demonstrate improved accuracy.^15^, ^16^, ^17^ Here, we used established EHR software tools and applied a free-text searching strategy to assess complications in surgical reports and postoperative clinical notes. This allowed us to analyze the safety profile of AH biopsies at large scale, involving 1418 cases that were performed during intraocular surgery by many surgeons in a variety of cases. We found the procedure to be safe, creating new opportunities for molecular disease analysis in living humans.

## Methods

### Study Approval

The study was approved by the Institutional Review Board of Stanford University and adhered to the tenets set forth in the Declaration of Helsinki. All patients underwent informed consent.

### Surgical Technique

**Figure 1:**
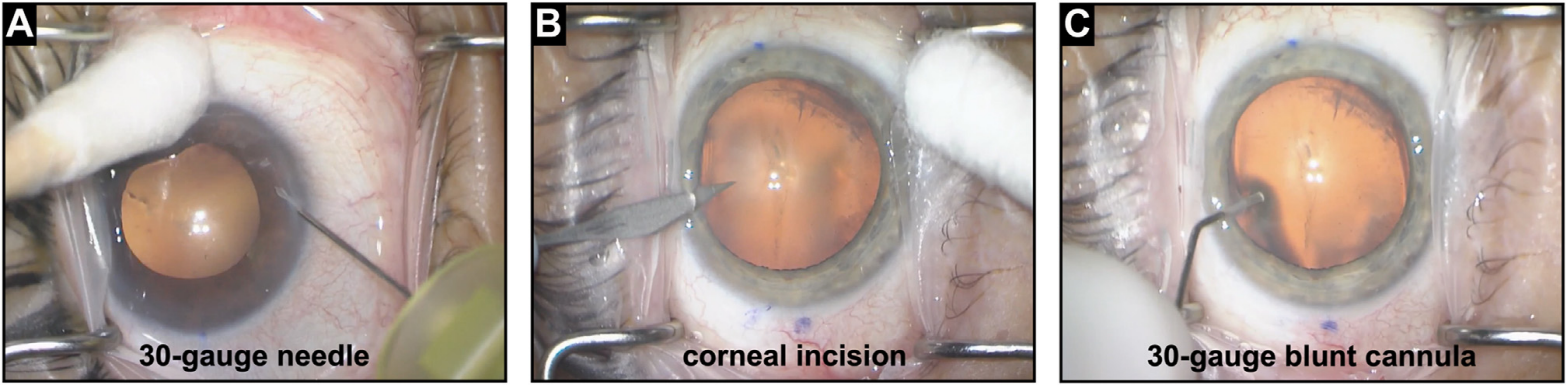
Two surgical techniques were applied to collect aqueous humor liquid biopsies during intraocular surgery. **A,** In technique 1, a 30-gauge needle connected to a 1-mL syringe was inserted perpendicular to the limbus and into the anterior chamber without prior incision. **B, C,** In technique 2, a 15° blade was used to make a corneal incision perpendicular to the limbus in the superotemporal quadrant (as part of the scheduled surgery), and an angled 30-gauge blunt cannula connected to a 1-mL syringe was inserted into the anterior chamber. In both techniques, 50 to 100 μL of undiluted aqueous humor was manually aspirated using the syringe.

Anterior segment or vitreoretinal surgeries were performed with either topical anesthesia using eye drops, peribulbar anesthesia with a sub-Tenon’s injection, retrobulbar anesthesia under monitored anesthesia care, or general anesthesia. Aqueous humor liquid biopsies were performed by 17 different ophthalmic surgeons and were all obtained using an operating microscope at the beginning of the surgery. Two techniques were followed based on surgeon preference (Fig 1). In the first technique, a surgeon inserted a 30-gauge needle connected to a 1-mL syringe perpendicular to the limbus and into the AC without prior incision. A total of 50 to 100 μL of undiluted AH was manually aspirated, as previously described.^18^ In the second technique, a 15° blade was used to make a corneal incision perpendicular to the limbus in the superotemporal quadrant, an angled 30-gauge blunt cannula connected to a 1-mL syringe was inserted into the AC, and 50 to 100 μL of AH was manually aspirated. In these cases, the corneal incision was part of the scheduled surgery. In both techniques, special attention was made to ensure the tip of the needle or the cannula remained over the peripheral iris in the mid AC to avoid damage to intraocular structures, including the corneal endothelium, iris, and lens. In vitrectomy cases, sclerotomies were created using a trocar-cannula system before the AC biopsy to ensure safe insertion of the trocars. After AH aspiration, the needle or the cannula were carefully removed from the AC, and syringes were passed to the scrub technician. Each surgeon only used one technique (needle or cannula) for all cases, and, therefore, we were able to determine the number of cases with each technique based on the number of cases of each surgeon. The fluid was expelled into a barcoded cryovial, and the vial was immediately transferred on dry ice in the operating room and then prepared for storage in a biorepository at −80°C.^18^ The case continued as per required for the primary surgical indication.

### Data Sources

The Stanford Ophthalmology biorepository stores human specimens that were collected during intraocular surgery at our institution.^18^ The biorepository is connected to a customized REDCap (Research Electronic Data Capture) database ^19^ that stores the patient identifiers and other metadata of each specimen (such as age, sex, disease, performed surgery, procedure date, laterality, and others).^18^^,^^20^ The sample annotation is performed immediately after sample collection and freezing in the operating room using a Mobile Operating Room Lab Interface.^18^^,^^20^ The Stanford Research Repository (STARR) cohort discovery tool is Stanford Medicine’s research patient data repository for clinical and translational research^21^ that allows us to capture the EHR data of all patients participating in the Stanford Ophthalmology biorepository study. The Stanford Research Repository provides access to information on patient demographics, diagnoses, surgical reports, and eye examination findings from each clinic visit.

### Assessing Operative Complications and Anesthesia Type Using EHR

**Figure 2:**
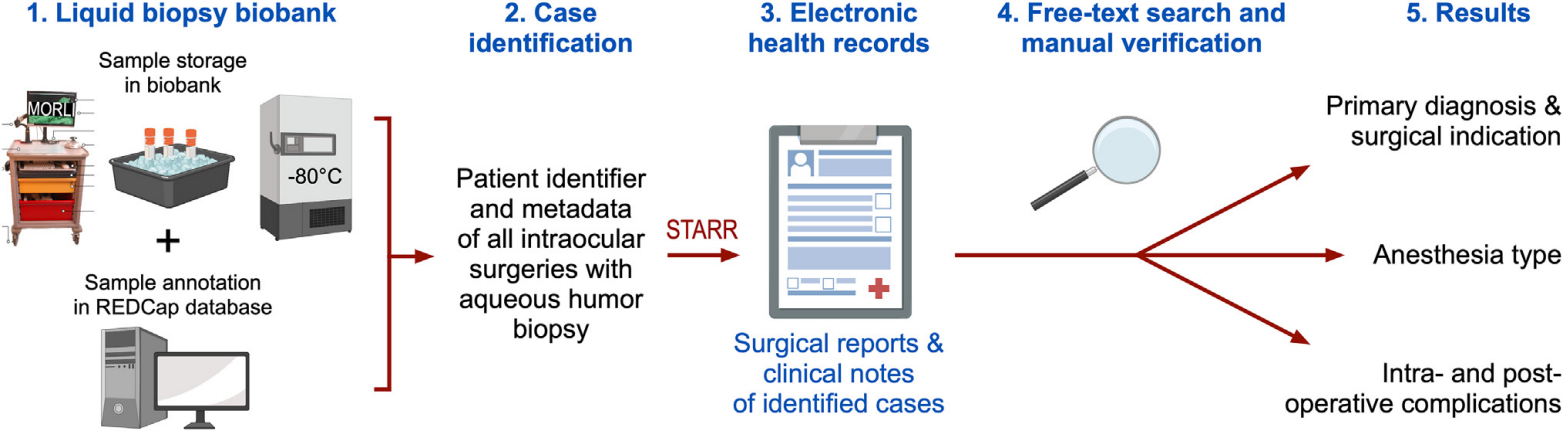
Electronic health record data analysis workflow. Our liquid biopsy biobank database was queried to identify patients that underwent intraocular surgery with aqueous humor liquid biopsy. The STARR tool was used to obtain electronic health records of these patients. We performed a free-text search with manual verification to determine the primary diagnosis and surgical indication, the anesthesia type, and intra- and postoperative complications. MORLI = Mobile Operating Room Lab Interface; REDCap = research electronic data capture; STARR = Stanford Research Repository.

The REDCap database of the Stanford Ophthalmology biorepository was queried to obtain a list of AH samples that were obtained during intraocular surgery at the Department of Ophthalmology at Stanford University between 2018 and 2023. The STARR tool was then used to review the EHR data of these cases. The Stanford Research Repository was queried to (1) confirm patient identifiers and procedure dates, (2) to extract the operative report (with matching procedure date) and all clinical notes for review, and (3) to extract the anesthesia text from the operative report (Fig 2). To assess the anesthesia type and intraoperative and postoperative complications, we considered all free-text entries in the operative reports and clinical notes. Using a free-text search, we developed an algorithm capable of checking for the presence or absence of a variety of anesthesia types and intraoperative and postoperative complications. All 17 surgeons were interviewed on the language they use in their operative reports and clinical notes to describe intraoperative and postoperative complications. Based on their response, the following terms were used for the free-text search: “lens touch,” “iris touch,” “cornea touch,” “bleeding”, “hemorrhage”, “hyphema,”, “tear”, “leak”, “shallow”, “movement”, “suprachoroidal”, and “endophthalmitis.” Our free-text search algorithm also checked for signs of negation (e.g., “no lens touch”) and did not flag such mentions as complication. The output of these analyses was a spreadsheet containing the original free-text by the clinicians along with binary flags (yes/no) for each of the abovementioned complications. Complications of the surgery that were not related to the AC biopsy were not tabulated for this study. A similar free-text search algorithm was developed to determine the anesthesia type of each case. The presence or absence of each of the following terms in the anesthesia text of the operative reports was assessed for each case: “topical,” “intracameral,” “subtenon,” “peribulbar,” “retrobulbar,” “lidocaine,” and “general.” The output was a spreadsheet with the original anesthesia free-text along with binary flags (yes/no) for each of the abovementioned anesthesia types. We also manually identified the preoperative ophthalmic diagnoses and the primary surgical indication and determined the time of follow-up for each case.

### Algorithm Validation

To validate our algorithm to assess for intraoperative and postoperative complications, 500 of the 1418 cases (35.3%) were randomly selected. The operative reports and chart notes were manually reviewed and compared to the binary flags provided by the algorithm. The reviewer was asked to take note of any misclassification of any complication by the algorithm. We then calculated the percentage of correctly classified complications as deemed by the reviewer. We also validated our algorithm to determine the anesthesia type used in each case. The anesthesia text was manually reviewed and compared to the binary flags provided by the algorithm. The reviewer checked each of the 1418 cases to ensure that the correct anesthesia type was selected, especially in those cases where more than 2 terms were found. All analyses were performed using R studio (version 2023.12.1 + 402, R version 4.3.2).

## Results

A total of 1418 AH liquid biopsies were obtained during intraocular surgeries. For 14 of these cases, there was a mismatch in the MRN, name, or date of birth fields between the REDCap database of the Stanford Ophthalmology biorepository and the STARR EHR data (less than 1% of cases). We found this was due to spelling errors that occurred during data entry in the operating room in the REDCap database, such as a missing leading “0” in the MRN or a wrong date of birth. We were able to manually confirm the correct identity of each case using a combination of all identifiers and with reference to the STARR data. Our free-text search algorithm accurately identified all intraoperative complications of the manually reviewed cases (100.0%). The accuracy for postoperative endophthalmitis was >99% (<1% false positive). The algorithm found 14 instances of the term “endophthalmitis” noted in patients EHRs. In all 14 instances, patients were seen for endophthalmitis before the AC tap, often years earlier, and there was no postoperative endophthalmitis following the AC tap. The algorithm accurately identified the anesthesia type in >93.6% of all 1418 cases. In 50 cases (3.5%), our search strategy did not find any hit. In those cases, none of the search terms or only imprecise information (e.g., “monitored anesthesia care”) was provided in the anesthesia section of the surgical report. A manual review of those cases revealed that the anesthesia type was provided in another section of the surgical report but not in the anesthesia section. We were able to determine the correct anesthesia type in each case. In 104 cases (7.3%), the term “general” was found using our free-text search, but our manual verification revealed that 40 of these cases (2.8%) were under local anesthesia combined with intravenous sedation. The reason for this discrepancy was that the term “general” was used imprecisely in the surgical report of these patients, most frequently as “transient general anesthesia,” although intravenous sedation was administered without any form of ventilation. We therefore manually classified these cases under the respective local anesthesia type. Overall, the free-text EHR search strategy was 100% error-free for surgical complications (500 cases reviewed), >99% for endophthalmitis (<1% false positive), and >93.6% for anesthesia type, and despite the need for manual examination, greatly increased the efficiency of the search. In combination with manual annotation, we were able to obtain the intended information for each of the 1418 cases.

The 1418 AH liquid biopsies were performed on 1154 patients by 17 experienced surgeons with 6 surgeons collecting ≥50 biopsies each. More than 85% of the samples were collected under topical (1021), peribulbar (125), or subtenon (61) anesthesia. Only about 15% of the cases (211) were performed under ocular muscle akinesia using retrobulbar (147) or general (64) anesthesia. In 65.7% of cases, a 30-gauge needle was used without prior incision, while, in the remaining 34.3% of cases, a blunt 30-gauge cannula was chosen, which was inserted following a corneal incision with a 15° blade. The most common primary surgical indications included cataract (711 specimens), cataract and glaucoma (207 specimens), glaucoma (147 specimens), retinal detachment (24 specimens), Fuchs endothelial dystrophy (21 specimens), and uveal melanoma (20 specimens). The remaining surgical indications are listed in Table 1. The intended volume of 50 to 100μL of undiluted AH was achieved in all cases.

**Table 1:** Aqueous Humor Liquid Biopsy Cases

	Diagnosis	Cases (n)	Prevalence (%)
Anterior segment	Cataract	711	50.0%
	Cataract, glaucoma	207	14.6%
	Glaucoma	147	10.4%
	Diabetic cataract	53	3.7%
	Cataract, pseudoexfoliation	11	0.8%
	Cataract, glaucoma, pseudoexfoliation	9	0.6%
	Diabetic cataract, glaucoma	9	0.6%
	Glaucoma, pseudoexfoliation	6	0.4%
	Cataract, retinitis pigmentosa	4	0.3%
	Cataract, proliferative diabetic retinopathy	3	0.2%
	Trauma	3	0.2%
	Uveitis, glaucoma	3	0.2%
	Cataract, corneal edema	2	0.1%
	Cataract, age-related macular degeneration	2	0.1%
	Retained lens fragments	2	0.1%
	Anterior segment-other	16	1.0%
Cornea	Corneal edema	38	2.7%
	Fuchs’ endothelial dystrophy	21	1.5%
	Failed corneal transplant	15	1.1%
	Corneal opacity	7	0.5%
	Cornea-other	8	0.6%
Retina	Retinal detachment	24	1.7%
	Epiretinal membrane	19	1.5%
	Proliferative diabetic retinopathy	11	0.8%
	Vitreous hemorrhage	9	0.6%
	Macular hole	8	0.6%
	Endophthalmitis	6	0.4%
	Retinal detachment, vitreous hemorrhage	4	0.1%
	Vitreous hemorrhage, proliferative diabetic retinopathy	4	0.3%
	Retinitis pigmentosa	3	0.2%
	Uveitis	3	0.2%
	Autosomal dominant neovascular inflammatory vitreoretinopathy	3	0.2%
	Macular hole, epiretinal membrane	2	0.3%
	Usher syndrome	1	0.1%
	Retina-other	22	1.6%
Tumor/Lesion	Uveal melanoma	20	1.4%
	Vascular lesion	1	0.1%
	Tumor/lesion-other	1	0.1%
Total		1418	100%

A list of primary diagnoses for surgical cases in which an aqueous humor liquid biopsy was performed for proteomic analysis.

Our analysis revealed that out of 1418 biopsies, there was only one minor complication (0.07%) with no long-term sequelae (Table 2). In the case of a 70-year-old male patient with a 2+ cataract in the right eye undergoing cataract surgery under topical anesthesia, a 30-gauge cannula was used to obtain an AH specimen following a paracentesis made with a 15° super sharp blade. On AH withdrawal, there was shallowing of the AC. Balanced salt solution on a 30-gauge canula was used to reinflate the AC, at which time a small Descemet membrane tear was noted adjacent to the paracentesis. The remainder of the surgery was performed without incident, and the tear remained localized and small without extension. On postoperative follow up over 3 months, there was no residual corneal edema or other sequelae associated with this tear. None of the other patients had any trauma to the lens or iris. No other complications, such as hyphema, entry site leak, AC shallowing forcing termination of fluid collection, suprachoroidal hemorrhage, or problems caused by patient’s movements were noted.

**Table 2:** Incidence of Complications With Aqueous Humor Liquid Biopsies (n = 1418)

Complication	Number (%)
Endophthalmitis	0 (0%)
Lens touch/trauma	0 (0%)
Iris touch/trauma	0 (0%)
Hyphema	0 (0%)
Descemet membrane tear	1 (0.07%)
Other corneal trauma	0 (0%)
Entry site leak	0 (0%)
Anterior chamber shallowing forcing termination of fluid collection	0 (0%)
Suprachoroidal hemorrhage	0 (0%)
Problems caused by patient’s movements	0 (0%)
Total	1 of 1418 (0.07%)

The median follow-up time at our institution was more than 7 months (223 days, range: 0–2003 days). Ninety-nine percent (1408) of the cases were seen for an immediate follow-up the next day; 97.1% (1377), 88.6% (1256), and 69.0% (978) were followed-up for 1 week, 1 month, and 3 months, respectively. There was no case of endophthalmitis following the AH biopsy.

## Discussion

In this study, we reviewed a large cohort of intraocular surgeries in which an AH liquid biopsy was performed regardless of the primary surgical indication and found only one minor complication related to obtaining the AH specimen, corresponding to a complication rate of 0.07%. Importantly, we did not observe any case of intraocular inflammation or endophthalmitis for all patients with 1 week follow-up (97.1%) and for all patients that had a 3-month postbiopsy follow-up (69.0%). These findings indicate that obtaining an AH biopsy during intraocular surgery using a 30-gauge needle or cannula is a safe procedure. Our study further demonstrates that EHR analyses that incorporate free-text searches are helpful to investigate intra- and postoperative complications, especially when examining operative reports that contain data not reflected in billing codes or “big-data” clinical repositories.

In smaller studies with mainly uveitis patients, AH biopsy was reported as a safe procedure when performed at the slit lamp in the outpatient clinic. Van der Lelij and Rothova^10^ investigated 361 AH biopsies obtained after preincision with a blade for diagnostic assessment in patients with uveitis. Apart from a small hyphema in 5 of 72 cases (6.9%), no other complications were reported. The largest study so far investigated 560 AH biopsies in patients with uveitis performed at the slit lamp without preincision and found 4 complications (0.7%), among them 1 case of a lens touch, 2 cases of air in the AC, and 1 case of an allergic reaction to the disinfectant.^12^ Because air in the AC is self-limiting and typically does not have significant consequences, the complication rate could be considered to be 1 of 560 cases (0.2%). Two other similar studies reported air in the AC in 2 of 70 cases (2.9%) ^11^ and no complication in 301 cases.^13^ Our findings in a cohort more than 2.5 times larger demonstrate that AH liquid biopsies can be safely obtained in the operating room across a variety of intraocular surgeries, ranging from corneal interventions to cataract and glaucoma surgery and to vitreoretinal procedures. One of the potential challenges of collecting AH samples at the slit lamp is that the patient can move the eye. During intraocular surgery in the operating room, retrobulbar and general anesthesia make the eye immobile, whereas under local anesthesia, the patient could still move the eye. In our study, >85% of the cases were under local anesthesia without ocular muscle akinesia, but no complications due to eye movements were observed. These findings suggest AC biopsies in an outpatient procedure room with an operating microscope and supine patient might reduce some complications reported when using an examination lane slit lamp with an upright patient. In addition, compared to AC taps performed as a stand-alone procedure at the slit lamp, sampling AH during intraocular surgery has the advantage that the risks generally do not exceed the risks of already planned intraocular surgery. Collecting these samples on a large scale could significantly enhance molecular analyses, including genomic, proteomic, and metabolomic studies, with the goal to improve our understanding of disease mechanisms in living humans.^1^, ^2^, ^3^, ^4^, ^5^, ^6^^,^^8^^,^^9^

In this study, 2 different surgical techniques were followed, one using a 30-gauge needle without prior incision and another using a blunt 30-gauge cannula that was inserted following a corneal incision with a blade. Using a 30-gauge needle has the advantage that the AC may be less likely to shallow, the wound is self-sealing, and the risk of blood contamination of the sample is minimized. Inserting a blunt cannula through the prior corneal incision that is generated as part of many intraocular surgeries has the advantage that no additional entry site must be created and that the risk of damage to intraocular structures may be lower compared to the first technique. Instead of the blunt cannula, a sharp 30-gauge needle may also be inserted through the corneal incision. However, with the 2 steps, the AC may be more likely to leak some fluid and shallow, thus the volume that can be collected could be lower. A single minor complication we observed was a small tear in Descemet membrane following insertion of the 30-gauge blunt cannula, but there was no serious impact on the rest of the case or on postoperative healing. Several insertions of the blunt cannula were also part of the normal surgery to deliver balanced salt solution and viscoelastic. Taken altogether, the results suggest that both techniques are safe and surgeon preference can be followed.

There are limitations to this study. Data was only included from one site, and the results may not be generalizable to other institutions. The purpose of our study was to determine complications associated with biopsy. Complications of the surgery unrelated to biopsy were therefore not reviewed. We performed a multi-instant search that included various EHR data for each patient, including the operative report and all clinical notes. Free-text searches are subject to spelling errors, but, even if a term to describe a complication was misspelled once, it is very unlikely that it was misspelled consistently in all documents that were searched. Importantly, our manual review confirmed that no complications were systematically missed. The anesthesia data had the highest inaccuracy. The reason was inaccurate use of terms to describe anesthesia type in the surgical report. Interviewing the anesthesia team to learn how the terms are used may improve the accuracy in future studies. Follow-up for some cases was not available beyond 3 months, and it seems unlikely that the AH biopsy before a complete intraocular surgery would trigger complications beginning after 3 months. Nonetheless, a prospective, randomized controlled trial analyzing all postoperative complications between cases with or without an AH biopsy would need to be conducted. Alternatively, a big data approach that reviewed EHR of tens of thousands of surgery cases might reveal an association with complications.

In conclusion, in this large cohort of 1418 AH liquid biopsies that were collected during intraocular surgeries demonstrated less than a 0.07% minor complication rate related to the sample collection itself. Our findings indicate that obtaining AH specimens during intraocular surgery is a safe procedure. Collecting these samples for research or in clinical trials could significantly improve molecular studies leading to a better understanding of disease pathophysiology in living humans. Our results further demonstrate that implementing free-text search in reviewing EHR could increase the size and quality of retrospective studies assessing surgical complications.
